# The Possibility of Plasma Membrane Transporters as Drug Targets in Oral Cancers

**DOI:** 10.3390/ijms26094310

**Published:** 2025-05-01

**Authors:** Chiharu Sogawa, Katsumitsu Shimada, Keisuke Nakano

**Affiliations:** 1 Department of Food and Health Sciences, Faculty of Environmental Studies, Hiroshima Institute of Technology, 2-1-1 Miyake, Saeki-ku, Hiroshima 731-5193, Japan; 2 Department of Clinical Engineering Faculty of Life Sciences, Hiroshima Institute of Technology, 2-1-1 Miyake, Saeki-ku, Hiroshima 731-5193, Japan; 3 Department of Clinical Phathophysiology, Matsumoto Dental University, 1780 Gobara Hirooka, Shiojiri 399-0781, Japan; 4 Department of Oral Pathology and Medicine, Faculty of Medicine, Dentistry and Pharmaceutical Sciences, Okayama University, 2-5-1 Shikata-cho, Kita-ku, Okayama-city, Okayama 700-8558, Japan

**Keywords:** SLC transporter, ABC transporter, oral cancer, oral squamous cell carcinoma

## Abstract

Plasma membrane transporters are increasingly recognized as potential drug targets for oral cancer, particularly oral squamous cell carcinoma (OSCC). These transporters play crucial roles in cancer cell metabolism, drug resistance, and the tumor microenvironment, making them attractive targets for therapeutic intervention. Among the two main families of plasma membrane transporters, ATP-binding cassette (ABC) transporters have long been known to be involved in drug efflux and contribute to chemoresistance in cancer cells. On the other hand, solute carriers (SLCs) are also a family of transporters that facilitate the transport of various substrates, including nutrients and drugs, and have recently been shown to contribute to cancer chemosensitivity, metabolism, and proliferation. SLC transporters have been identified as potential cancer biomarkers and therapeutic targets, and their expression profiles suggest that they could be utilized in precision oncology approaches. We summarize previous reports on the expression and role of ABC and SLC transporters in oral cancer and discuss their potential as therapeutic targets.

## 1. Introduction

Plasma membrane transporters play a crucial role in the uptake of substances into cells and the excretion of substances from cells, thus representing a significant class of functional proteins. These membrane proteins are mainly classified into two types: solute carriers (SLCs) and ATP-binding cassette (ABC) transporters. The ABC transporter is bound to ATP, and it utilizes the energy derived from ATP to facilitate the active transport of substances from the intracellular space to the extracellular environment. Consequently, some cancer cells employ ABC transporters to facilitate the release of chemotherapeutic drugs from the cell, thereby diminishing the therapeutic efficacy of these drugs [1,2,3]. In contrast, the SLC transporter does not require ATP and primarily facilitates the entry of small molecules into the cell via a distinct mechanism. SLCs play a pivotal role in the process of drug uptake by cancer cells. These transporters regulate the transport of various molecules across the cell membrane, thereby impacting the efficacy of drugs and the progression of cancer. Additionally, SLCs have been implicated in cancer cell metabolism and are increasingly recognized as potential drug targets [4].

Cancers that are collectively referred to as head and neck cancers usually develop in the squamous epithelium that covers the mucous surfaces of the head and neck (for example, the mouth, throat, and vocal cords). Head and neck cancers can also arise from the salivary glands, paranasal sinuses, or muscles and nerves of the head and neck, but these types of cancers are much less common than squamous cell carcinoma [5,6]. The majority of head and neck cancers are diagnosed as cancers of the mouth, throat, or vocal cords. Cancers of the paranasal sinuses and nasal cavity and salivary gland cancers are much rarer. Oral cancer is the most common cancer of the head and neck. Oral squamous cell carcinoma (OSCC) accounts for about 90% of oral cancers, has a low survival rate, and tends to metastasize and recur. Tobacco, alcohol, and human papillomavirus (HPV) have been identified as contributing factors to OSCC, with both genetic and non-genetic elements being implicated in its development [7,8]. Current therapeutic interventions for OSCC are associated with substantial adverse effects and complicate patient management. Despite recent therapeutic advancements, the prognosis for patients with OSCC remains dismal, underscoring the need for early diagnosis and the identification of molecular pathways. SLC and ABC transporters play a critical role in the transportation of diverse molecules across cell membranes. These transporters influence the efficacy of antineoplastic drugs and the progression of cancer due to their involvement in cancer cell metabolism. The hypothesis that transporters are viable therapeutic targets in oral cancer is supported by several studies that have demonstrated their involvement in drug resistance mechanisms and their expression in cancer cells [7,9]. This review outlines the expression of plasma membrane transporters such as SLC and ABC transporters and their role as therapeutic targets in cancer treatment, including findings in OSCC and head and neck squamous cell carcinoma (HNSCC).

## 2. ABC Transporters

The ABC transporters are classified into seven subfamilies, ABCA through ABCG, with 48 genes reported [10]. A typical ABC transporter consists of two nucleotide-binding domains (NBDs) and two transmembrane domains (TMDs). The TMDs are composed of a series of six to eleven α-helixes that traverse the lipid bilayer. The region between these α-helixes constitutes the substrate binding site, which can be either intracellular or extracellular, depending on the specific function being performed. Two TMDs bind to each other, thereby forming transmembrane sites within the molecule. These transmembrane sites serve to determine substrate specificity. The two NBDs face the cytoplasmic side. In addition to the Walker A and Walker B motifs, each NBD contains a highly conserved signature motif (also known as the C motif) specific to ABC transporters, which are collectively referred to as ATP-binding cassettes. The mechanisms by which ABC transporters facilitate molecular transport are not yet fully elucidated, but current hypotheses posit that these processes occur through alterations in the higher-order structure of the protein during each stage of ATP binding, hydrolysis, and dissociation. It is important to note that each of these transport mechanisms is characterized by a distinct substrate and function [11,12,13,14]. In the following, we discuss ABC transporters that have been reported to be associated with drug efflux and cancer stem cells, particularly in oral cancer. Table 1 lists the ABC transporters.

### 2.1. Drug Excretion from Cancer Cells by ABC Transporters

ABC transporters are known to affect and reduce the efficacy of chemotherapy treatments by expelling drugs from cancer cells. ABCB1 was the first ABC transporter identified and was named p-glycoprotein because it is a glycoprotein involved in the membrane permeabilization of substances. ABCB1 has also been reported as a factor in multidrug resistance 1 (MDR 1) for multidrug resistance to anticancer drugs [15,16,17,18]. A wide range of pharmaceutical agents are transported by human ABCB1, including anticancer drugs [3,14,19,20]. In oral cancer, overexpression and genetic mutations of ABCB1 have also been reported and are thought to be among the causes of reduced efficacy of chemotherapeutic agents [21,22,23]. In addition, ABCC1 and ABCG2 are well-studied molecules found in drug-resistant tumors. In OSCC, ABCC1 and ABCG2 were increased 1.6- and 2.1-fold, respectively, in OSCC specimens from chemotherapy-treated patients [24]. ABCC1, also known as MRP1, has been involved in drug elimination in various tissues of the body, including oral cancer, like ABCB1 [19,20,21,25,26,27]. ABCG2, also known as breast cancer resistance protein (BCRP), was isolated as a gene highly expressed in resistant breast cancer cells [28,29,30,31]. In addition, because they often confer resistance to multiple anticancer drugs at the same time, they have caused multidrug resistance in cancers, including oral cancer, which has been considered clinically problematic [20,31,32,33,34].

Other ABC transporters, ABCA1, ABCC3, ABCC5, and ABCC10, have also been reported to be capable of conferring anticancer drug efflux and resistance [14,26,35,36]. ABCC3 and ABCC10 were found to be expressed in a variety of human tissues. ABCC3 is expressed especially in the liver, intestine, and adrenal glands, while ABCC10 is detected in tissues such as the pancreas, testis, and colon. ABCC3 was also suggested to be associated with drug resistance in oral cancer [24]. ABCC5 has also been reported to play an important role in MDR by reducing the intracellular concentration of drugs and their efficacy through ATP-dependent efflux [36]. ABCG family member ABCG1 functions in intracellular cholesterol transport and homeostasis [37]. ABCG1 gene amplification was observed in 10–35% of metastatic cancers and was highly expressed in metastatic colorectal cancer. In addition, it was reported that ABCG1 depletion suppressed the release of extracellular vesicles from cancer cells, inducing lipid accumulation and inhibiting cancer cell proliferation [38].

**Table 1:** List of ATP-binding cassette (ABC) transporters expressed in oral cancer.

Family Name	Approved Symbol	Alias Symbol	HGNC ^#^ ID	References
ABCA (ABC I family)	ABCA3	ABC-C	33	[39]
ABCB (MDR/TAP family)	ABCB1	P-gy (MDR1)	40	[3,14,18,19,20,21,22,23,40,41]
	ABCB4	MDR2	45	[39]
	ABCB5	EST422562	46	[3,42]
	ABCB10 *	EST20237	41	[43]
ABCC (CFTE/MRP family)	ABCC1	MRP1	51	[19,20,21,25,26,27,40,41]
	ABCC2	MRP2	53	[44]
	ABCC3 *	MRP3	54	[26]
	ABCC5 *	MRP5	56	[36]
	ABCC10 *	MRP7	52	[26]
ABCE (OABP family)	ABCE1	OABP	69	[45]
ABCG (WHITE family)	ABCG1 *	ABC8	73	[38]
	ABCG2	BCRP	74	[19,20,21,27,29,30,31,32,33,34,40,41,44]

* Not reported in OSCC and HNSCC. ^#^ Human Gene Nomenclature Committee.

### 2.2. Cancer Stem Cells and ABC Transporters

Cancer stem cells (CSCs) are known to be involved in important processes such as carcinogenesis, tumor invasion, metastasis, and resistance to various therapies, including chemotherapy. ABC transporters play an important role in protecting CSCs from drug-induced damage. CSCs express high levels of ABCB1, ABCC1, and ABCG2, and these transporters are involved in the efflux of chemotherapeutic drugs, reducing intracellular drug concentrations and contributing to the drug-resistant phenotype [46]. ABCG2 is expressed at high levels. This high expression is a distinguishing feature of CSCs compared to other cells in the tumor and contributes to their drug-resistant nature [28]. Cancer stem-like side population (SP) cells, which are responsible for resistance to chemotherapy drugs and tumor recurrence, have been identified in a variety of tumors. SP cells highly express ABCB1 and ABCG2 and can be characterized by Hoechst 33342 dye exclusion by FACS [47]. It was suggested that ABCG2 and ABCB1 play a protective role for CSCs by expelling biological foreign substances from the cells and that they are involved in both the maintenance of the undifferentiated state and the proliferative potential of stem cells [48]. Muriithi et al. reported that ABCB1, ABCB5, and ABCG2 are involved in the regulation of cytokine signaling, which is essential for the maintenance of CSCs. These transporters help release porphyrins and reduce oxidative stress, contributing to CSC survival and maintenance [3]. ABCB2 and ABCB3 are associated with the downregulation of antigen-presenting mechanisms and help CSCs evade immune mechanisms [3].

### 2.3. OSCC and ABC Transporters

Cisplatin, an alkylating agent, induces DNA damage by forming cisplatin-DNA adducts, leading to cell cycle arrest and apoptosis. Cisplatin-based chemotherapy or chemoradiotherapy is considered first-line therapy in the treatment of advanced OSCC. However, only some patients with OSCC benefit from cisplatin therapy due to resistance. The regulatory role of ABC transporters in cisplatin-resistant OSCC is still poorly understood. There have been several reports of elevated ABCG, ABCB1, and ABCC1 gene expression in cisplatin-resistant OSCC cell lines [40,41]. Furthermore, it was shown that the Hedgehog signaling pathway promotes multidrug resistance of OSCC by regulating the ABC transporter [49]. The well-known tumor suppressor gene p53 is commonly mutated in human cancers. p53 mutations also lead to cancer’s resistance to treatment. Mutations in p53 promoted the upregulation of ABCC2 and ABCG2 and increased their metabolic activity [44]. CD44-positive cells in OSCC stem cells were found to express ABCB1, and cisplatin-resistant FaDu cells exhibit stem cell-like characteristics and high ABCB1 expression, highlighting its role in chemotherapy resistance [22]. Furthermore, ABCB5 was found to be expressed in CD44-positive cancer cells, indicating that it is a CSC marker in OSCC [42]. Several signaling pathways, including the Wnt/β-catenin pathway, were reported to be involved in the regulation of ABC transporter expression in CSCs [20,40]. It was concluded that Circ-ABCB10 is significantly upregulated in OSCC and is closely associated with staging and distant metastasis in patients with OSCC [43]. ABCE1 expression was found to be low in adjacent non-tumor tissues but high in oral cancer; there was an inverse relationship between ABCE1 expression and the degree of cancer cell differentiation [45]. Zahra et al. showed that ABCF3 is a common target of miRNAs that serve as biomarkers for oral cancer [50]. Recent findings have reported that the upregulation of ABCA3, ABCB1, and ABCB4 contributes to OSCC resistance to TNF-mediated cell death [39]. The combination of ABC transporter inhibitors and Smac mimetic, a small molecule inhibitor that antagonizes inhibitor of apoptosis proteins (IAPs), was expected to improve sensitivity to cell death [39].

## 3. SLC Transporters

Currently, 446 molecules from 66 families of SLCs have been reported. Most are expressed on the plasma membrane, but some are expressed on the membrane of mitochondria or other intracellular organelles. Advances in structural biology techniques such as X-ray crystallography and cryo-electron microscopy have enabled high-resolution structure determination of SLC transporters [51]. SLC transporters are generally comprised of multiple transmembrane helixes that constitute a core structure extending across the lipid bilayer. These helices serve as a conduit for substrate movement across the membrane. The number of transmembrane segments varies among SLC families; however, they are predominantly characterized by multi-pass transmembrane domains comprising 12 to 14 helixes [52]. Each SLC transporter can function as a symporter, antiporter, or uniporter. Symporters use ion gradients as a driving force to co-transport substrates with ions such as H^+^ and Na^+^. Antiporters exchange one substrate for another across the membrane. These maintain intracellular ion concentrations, and electrogenic transporters can generate a net charge across the membrane. Antiport involves substrate transport against a concentration gradient but uses the energy generated by the transport of the other substrate down the concentration gradient. Uniporters facilitate the passive transport of substrates down the concentration gradient [51,53,54,55]. SLC transporters are responsible for the transport of a variety of substrates, including ions, amino acids, sugars, lipids, and drugs. This diversity is reflected in the structural variation among the different SLC families.

The relevance of SLC transporters to cancer has not been investigated as much as ABC transporters. However, the importance of SLCs in cancer has been gaining attention in recent years. Previous studies have reported that the SLC superfamily is involved in various steps of tumorigenesis, including proliferation, apoptosis, invasion and metastasis, chemotherapy resistance, and other cancer-related processes [4]. SLC transporters are essential for the uptake of glucose, amino acids, and other nutrients essential for cancer cell survival and growth. SLC transporters are frequently found to be overexpressed in cancer cells. This increase in expression is driven by the elevated metabolic demands associated with accelerated cell growth and division [56,57,58,59,60,61,62]. In addition, SLC transporters are essential for the transport of anticancer drugs into the cell, and their expression can have a significant impact on the efficacy of cancer therapy. On the other hand, the potential of SLC transporters to serve as biomarkers for cancer diagnosis and prognosis has also been explored, with some transporters showing promise as early diagnostic markers for oral cancer [63,64]. Table 2 lists the SLC transporters.

### 3.1. Glucose Transporters

The SLC2 family, called the glucose transporter family, is responsible for energy-providing glucose translocation into the cell. Overexpression of SLC2A1 (GLUT-1) is a common feature of human cancers, including OSCC. Whereas normal tissues show limited expression of SLC2A1, OSCC shows a significant increase [57,70,71,73,74]. High expression of SLC2A1 promotes increased glucose uptake required for tumor growth and survival under hypoxic conditions. Cancer cells also exhibit the Warburg effect and utilize high glucose levels for energy regardless of oxygen. The increased glycolytic activity due to SLC2A1 expression is associated with the Warburg effect [57,75,76]. MicroRNA-218 regulates SLC2A1 and glucose metabolism. The inhibition of miRNA-218 increased SLC2A1, which affected glucose metabolism and promoted OSCC growth [77]. The HIF-1 signaling pathway was a key regulator of high glycolytic metabolism in OSCC and enhanced expression of glucose transporters [78]. Using SLC2A1 overexpression in tumors, SLC2A1 expression combined with imaging techniques such as FDG-PET can assess tumor metabolism and predict patient outcomes [71]. Furthermore, SLC2A1 expression has been shown to be increased in the early stages of OSCC development and correlated with the clinical stage and pathologic grade, invasion, resistance to treatment, and survival [57,58,102,103,104,105]. These reports suggest that SLC2A1 may be an important factor in determining the prognosis of OSCC.

In addition to SLC2A1, SLC2A2 and SLC2A4 were also highly expressed in OSCC [70,79]. In addition, cancer cells often use fructose as an alternative energy source. In lung tumors, for example, high expression of SLC2A5 allows fructose to be used even when glucose is in short supply [61]. Knockdown of SLC2A5 inhibited fructose uptake and utilization and reduced tumor cell growth. Furthermore, a positive correlation was found between SLC2A5 and IL-6 expression in a variety of cancers. This process involves the transcription factor STAT3, which binds to the SLC2A5 promoter region and increases transcription of SLC2A5 in response to IL-6 [84]. Hypoxic conditions promoted SLC2A5 expression, fructose uptake, and cancer cell survival. Pharmacological inhibitors of SLC2A5 have been shown to be effective in a variety of cancers [61].

SLC2A3 was also overexpressed in more than 20% of OSCC cases [80]. Recent studies have identified a correlation between high expression of SLC2A3 and various biological processes, including epithelial–mesenchymal transition (EMT), angiogenesis, hypoxic pathways, and NF-κB signaling pathways. These findings suggest a potential role for SLC2A3 in the adaptation and survival of cancer cells in adverse environments [81,82]. SLC2A3 expression was found to be negatively correlated with immune cells, indicating that high SLC2A3 expression predicts poor prognosis in patients with HNSCC [82]. Overexpression of SLC2A3 also increased OSCC lactate production and glucose consumption, promoted survival and proliferation, and altered EMT. It also activated the TGF-β signaling pathway. Lactate production from SLC2A3 promoted tumor growth and invasion, highlighting the SLC2A3/LA/TGF-β regulatory axis in OSCC [83].

El-Gebali et al. reviewed the role of the SLC transporter family in cancer as it applies to cancer hallmarks proposed by Hanahan et al. [4,106]. They reported that SLC2A1, SLC2A3, and SLC2A4 are correlated with angiogenesis in ovarian cancer [4]. The H⁺-myoinositol transporter SLC2A13 has been reported as a CSC marker for OSCC [4,85]. SLC5A1 is a sodium-glucose co-transporter (SGLT), one of the transporters essential for glucose uptake into the cell. SLC5A1 is overexpressed in a variety of cancers, including liver, colon, and prostate cancer, but no significant differences were found between OSCC and normal oral keratinocytes [57].

### 3.2. Amino Acid Transporters

The SLC transporter superfamily of amino acid transporters is important for maintaining amino acid homeostasis in cells and has been implicated in various types of cancer, including oral cancer. These transporters are involved in cancer cell proliferation, metabolic reprogramming, and cell death by promoting the uptake of essential nutrients and metabolites required for tumor progression [67,107]. In HNSCC, SLC1A5, SLC7A5, and SLC7A11 were found in 59%, 61%, and 21% of patients, respectively, and SLC1A5 and SLC7A5 expression was significantly associated with staging, lymph node metastasis, lymphatic infiltration, and cell proliferation, while SLC7A11 expression was significantly associated with advanced stage and tumor factors [65]. SLC1A5, also called ASCT2, is a neutral amino acid transporter and transports glutamine. Its expression has been reported to be upregulated in a variety of cancers [67,68]. Zheng et al. reported that SLC1A5 expression is not associated with prognosis but is associated with the exclusion of CD8-positive T cells in the tumor microenvironment. Furthermore, dysregulation of SLC1A5 is associated with tumor growth and with increased resistance to apoptosis. SLC1A5 promotes ATP production and mTORC1 activity in mitochondria [66,67]. The inhibition of SLC1A5 decreases glutamine uptake and inhibits cancer growth. On the other hand, oxidative stress was increased, as was sensitivity to anticancer drugs such as cisplatin [67]. SLC1A5 functionally couples with SLC7A5 and promotes mTOR activation in cancer cells [68,69]. SLC7A5 (LAT1) is a branched-chain amino acid transporter that binds to CD98 to aid in its localization to the plasma membrane. The high expression of SLC7A5 mRNA is found in a variety of human tissues [4,62,87,93]. High expression of SLC7A5 mRNA is also found in HNSCC [82]. SLC7A5 activates the Akt/mTOR pathway to immunosuppress the tumor microenvironment and enhance radioresistance of cancer [67]. The SLC7A11/SLC3A2 complex (system X_c_^−^) is a cystine/glutamate antiporter that is frequently overexpressed in cancer cells, including oral cancer. It promotes cystine uptake for glutathione biosynthesis and protects cells from oxidative stress and ferroptosis [94]. Furthermore, upregulation of SLC7A11 promotes disulfidosis in OSCC [62,87,92,95].

SLC3A2 is a neutral amino acid transporter that interacts with other transporters and supports amino acid uptake and tumor growth in cancer [62,87]. The SLC3A2 gene is important for disulfide production and metabolism and affects tumor invasiveness; overexpression of SLC3A2 led to decreased expression of PD-1 and CTLA-4 in SCC-9 cells. The role of SLC3A2 in immune evasion and tumor progression highlights its potential as a therapeutic target. Future studies should focus on the interaction between SLC3A2 and immune responses in OSCC therapy [86].

Other transporters involved in glutamine metabolism, SLC38A1 and SLC38A5, have been implicated in the uptake of glutamine, which is essential for cancer cell metabolism and growth [59]. The upregulation of SLC38A1 expression has been reported in OSCC [101]; SLC38A1, also known by various names such as SNAT1, SAT1, SA2, and ATA1, has been identified as a plasma membrane transporter that plays an important role in the intracellular accumulation of glutamine. SLC38A1 was upregulated in OSCC, suggesting a role in the uptake of glutamine, which is essential for cancer cell metabolism and proliferation [101].

### 3.3. Other SLC Transporters

SLC16 family transporters (MCTs) regulate monocarboxylic acid transport and influence cancer metabolism. They also play a role in lactate export and pH regulation. These transporters help maintain an acidic microenvironment favorable to cancer progression and are considered promising targets for cancer therapy [108]. MCTs are overexpressed in many cancers and are associated with proliferation [61,62,87]. SLC16A3 (MCT4) has been shown to regulate cell migration and invasion, particularly in breast cancer cell lines, by interacting with β1 integrin and altering focal adhesion [4]. SLC16A3 is associated with oral cancer progression and poor prognosis in head and neck cancers, including OSCC [59]. It was significantly upregulated in patients with HNSC and associated with poor prognosis [82]. Inhibition of MCT4 was shown to potentially reduce cancer progression [63]. MCT4 inhibitors are associated with EMT, angiogenesis, hypoxia, and other processes. They are associated with important cancer pathways, and ongoing research on MCT4 inhibitors suggests their potential therapeutic significance.

The SLCO family is an organic anion transporter polypeptide, previously classified as the SLC21 family. SLCO1A2, SLCO1B3, and SLCO2A1 showed increased expression in HNSCC compared to normal tissue. In contrast, SLCO4A1 decreased. SLCO1A2 was significantly upregulated in breast cancer [62]. SLCO2B1 was found to be a significant predictor of 5-year survival in patients with HNSCC [97]. SLCO1B1 and SLCO1B3 promoted paclitaxel uptake; SLCO2A1 was associated with increased prostaglandin levels and supported cancer stem cell-like appearance in breast cancer [62]. Regulation of the SLCO family may lead to the prevention of drug resistance in many cancers [93,96].

SLC22A1–3 is an organic cation transporter (OCT) family member that is also involved in the transport of anticancer drugs. Cisplatin and oxaliplatin are substrates of SLC22A1 (OCT1). The role of OCT1 in the transport of imatinib was crucial for the treatment of chronic myeloid leukemia [96]. OCT1 expression may serve as a biomarker for personalized cancer therapy. Other anticancer drugs such as irinotecan and paclitaxel are also affected by OCT1. Patients with HNSCC with high SLC22A3 (OCT3) expression had significantly longer survival compared to patients with low expression in advanced-stage HNSCC receiving cisplatin-assisted therapy. This result was supported by the fact that SCC-4 cells overexpressing SLC22A3 showed increased uptake of cisplatin and enhanced cytotoxic effects [98]. In addition, the organic cation/carnitine transporters (OCTNs) SLC22A4 (OCTN1) and SLC22A5 (OCTN2) are involved in the transport of many drugs, including cisplatin, oxaliplatin, and imatinib. SLC22A16 (OCT6, CT2) was also involved in the uptake of doxorubicin and bleomycin, and its expression affected the drug sensitivity of leukemic cells [96]. Although these genes have not been studied in detail in oral cancer, certain cancer cell lines show high expression of SLC22A4 and SLC22A5 [93,96]. These gene mutations also affect drug sensitivity and the appearance of side effects. In addition, SLC22A5 and SLC22A16 affect survival and treatment response in patients with lung cancer [109]. Specific differences between lung adenocarcinoma (LUAD) and lung squamous cell carcinoma (LUSC) subtypes suggest that these genes may serve as biomarkers of patient outcome and influence targeted therapy in lung cancer treatment. In summary, OCTNs are potential therapeutic targets among patients with cancer [110]. 

The SLC28 and SLC29 subfamilies are involved in gemcitabine transport and resistance mechanisms [92]. SLC29A1 (ENT1) has been implicated in the uptake of antimetabolites, and although high expression of SLC29A1 has not been reported in oral cancer, its expression level could be associated with response to 5-FU, specifically in colorectal cancer [62].

The SLC30 and SLC39 families are the two main families that transport zinc; SLC39 facilitates zinc uptake into the cytoplasm, while SLC30 is responsible for zinc efflux within cell organelles [99,111]. They play opposing roles in zinc homeostasis affecting cancer growth. In OSCC, the SLC30 and SLC39 families have not been fully investigated. In some cancers, downregulation of the SLC39 family is often reported to occur, resulting in decreased intracellular zinc levels. This reduction in zinc can protect malignant cells from the cytotoxic effects of high concentrations of zinc commonly found in normal cells [99,100]. Particularly, changes in SLC30A1 (ZRC1) expression are associated with impaired zinc homeostasis in cancer. Loss of function by mutations in SLC30A1 may reduce intracellular zinc levels and contribute to cancer progression [99]. In addition, SLC39A4 (ZIP4) and SLC39A6 (ZIP6) are involved in angiogenesis and EMT and promote cancer metastasis [4,92]. SLC39A4 and SLC39A13 (ZIP13) have been implicated in ovarian cancer progression and metastasis. Furthermore, SLC39A13 was shown to affect the chemosensitivity of cancer cells to certain drugs [87]. SLC31A1 also regulates copper homeostasis and is associated with platinum-based drug resistance in ovarian cancer [87].

The SLC6 family is a family of transporters that transport neurotransmitters. Although not reported in oral cancer, it has been reported as a transporter associated with the growth of several other cancers. SLC6A3 is a well-known dopamine transporter, and studies indicate that SLC6A3 expression changes in kidney and colorectal adenocarcinomas may be a marker of cancer malignancy and that SLC6A3 gene changes affect cancer prognosis [88,89,90]. Furthermore, we reported on the metastasis-inhibitory effects of drugs targeting SLC6A3 [90]. SLC6A8 is called a creatine transporter, and RGX-202, a therapeutic agent that inhibits SLC6A8, inhibits the progression of colorectal cancer metastasis, indicating that creatine metabolism may be a therapeutic target [91]. SLC6A14 is a neutral and cationic amino acid transporter that contains glutamine. It is one of the amino acid transporters that has not been reported in oral cancer but is upregulated in invasive cancer [68,112]. The inhibition of SLC6A14 causes amino acid deficiency and inhibits tumor growth. SLC6A14 inhibition induces autophagy and inhibits prostate cancer cell growth [92].

**Figure 1:**
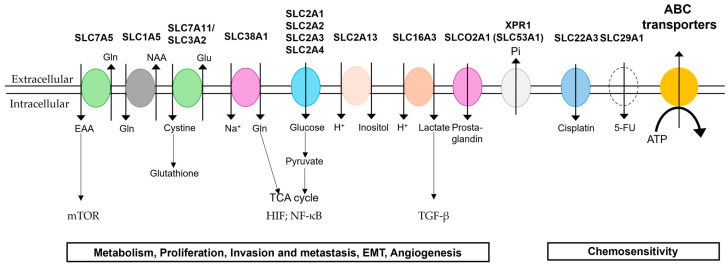
Schematic representation of the major membrane transporters expressed in oral cancer. SLC1A5, SLC7A5, SLC7A11/SLC3A2, SLC38A1, SLC2A1–4, SLC2A13, SLC16A3, and SLCO2A1 are involved in metabolism, proliferation, invasion and metastasis, EMT, and angiogenesis. SLC22A3, SLC29A1, and ABC transporters are involved in chemosensitivities. Uniporters: XPR1(SLC53A1); SLC2A1–4; SLCO2A1. Antiporters: SLC1A5; SLC7A5; SLC7A11. Symporters: SLC2A13; SLC16A3; SLC38A1. EAA, essential amino acids; NAA, neutral amino acids.

In addition, Cho et al. used databases (The Cancer Genome Atlas (TCGA) and Human Protein Atlas (HPA)) to comprehensively evaluate the expression changes of the SLC transporter family in HNSCC and their prognostic significance [82]. As a result, they listed SLC2A3 and SLC16A3 as well as SLC25A32 and SLC53A1 as therapeutic targets. SLC25A32, the gene encoding the mitochondrial folate transporter (MTF), is essential for mitochondrial folate transport and is presumed to be an essential transporter for cancer cell metabolism and growth; it is reported to be involved in phosphate efflux and tumorigenesis in ovarian cancer [87]. SLC34A2 is upregulated in ovarian cancer, affecting phosphate balance and correlating with decreased survival [87]. The SLC transporters expressed on the plasma membrane of mainly OSCC and/or HNSCC are summarized and schematically illustrated in Figure 1.

## 4. Plasma Membrane Transporters as Potential Drug Targets

The investigation of plasma membrane transporters is essential to understanding the mechanisms by which drugs and nutrients influx and efflux cancer cells. Understanding these transporters can help us understand why therapeutics are effective in some cancers and not in others. Many ABC transporters are found in cancer cells, which can cause drugs to be ejected and make treatment difficult. The inhibition of ABC transporters has been the focus of research to overcome drug resistance in cancer therapy. The use of modulators of ABC transporters could increase the effective intracellular concentration of anticancer drugs, potentially overcoming drug resistance and improving therapeutic outcomes [1]. In addition, the development of drugs that specifically target the ABC transporter of CSCs can enhance the delivery and efficacy of chemotherapeutic agents. Several strategies are being explored to inhibit the ABC transporter, including the use of small molecule inhibitors. However, these have shown limited success in clinical trials due to compensatory mechanisms and toxicity issues [93]. ABC transporter inhibitors have been investigated to address drug resistance caused by ABC transporters. Many first- and second-generation ABC transporter inhibitors were withdrawn from clinical trials due to high toxicity [113]. Toxicity was attributed to the fact that these inhibitors do not act specifically on cancer cells but also affect normal cells, and to interactions with other drugs, especially those metabolized by CYP450 enzymes [113]. Tariquidar is a third-generation inhibitor that can reverse the resistance of doxorubicin and vinblastine in advanced breast cancer. However, tariquidar was found to be toxic in a phase III clinical trial for non-small cell lung cancer (NSCLC) [20]. Many tariquidar analogs have been synthesized to optimize their pharmacological properties. Its new derivatives are expected to have low toxicity and high efficacy [93]. Advances in genomic diagnostic methods suggest that ABC transporter resistance may be restricted to specific subsets of tumors or patient populations, and the limited clinical trial results may be due to study design flaws that do not account for differences in ABC transporter tumor expression in patients [31].

Tyrosine kinase inhibitors (TKIs), on the other hand, have been identified as inhibitors of ABC transporters that can increase the cytotoxicity of chemotherapeutic agents by preventing drug efflux [114]. Strategies to overcome drug resistance in cancer include inhibition of ABC transporters that prevent the excretion of anticancer drugs as well as targeting SLC transporters to enhance drug delivery. In addition, they include the development of prodrugs and nanocarriers that utilize SLC transporters and the regulation of transporter expression to improve drug uptake [93,96,115]. However, challenges remain in understanding the complex interactions between transporters and drugs, and the variability in transporter expression and function across cancer types and stages adds to this complexity [59,115]. Future research should focus on characterizing the expression patterns of SLC transporters in oral cancer and developing personalized medicine approaches to optimize drug delivery and therapeutic efficacy considering these patterns [82,116].

High expressions of transporters such as glucose and amino acids in tumors allow targeted drug delivery to tumor cells. Nanoparticles targeting SLC transporters enhance tumor imaging and drug delivery [60,117]. Targeting glucose metabolism via SLC2A1 is therapeutically promising because it can inhibit OSCC progression. New imaging probes such as WZB117-IR820 targeting SLC2A1 are now being developed to enhance early detection and complete tumor resection [118]. In addition, SLC2A3 is another potential target for inhibiting OSCC progression as a molecule involved in glucose metabolism in oral cancer [82]. In addition, SLC16A3, which exports excess lactate and protons along with ABC transporters, could also be a target for inhibiting OSCC progression [108]. Preclinical studies have shown that SLC2 (GLUT) inhibitors can potentiate the effects of radiation therapy. For example, 2-deoxy-D-glucose has been reported to downregulate SLC2A1 expression in OSCC cell lines and enhance the effects of radiation therapy [57]. Inhibition of SLC2A1 is associated with increased apoptosis of cancer cells, suggesting a potential role in overcoming resistance to chemotherapeutic agents such as cisplatin [57]. However, clinical studies have been less conclusive regarding their efficacy. Variations in SLC2A expression may affect the efficacy of GLUT inhibitors and complicate their clinical application [57,119]. JPH203, an SLC7A5 (LAT1) inhibitor, which is also highly expressed in HNSCC, has completed phase II clinical trials in advanced biliary tract cancer and has shown promise in the prevention of biliary tract cancer [67]. SLC16 family transporters (MCTs), which export excess lactate and protons along with ABC transporters, could also be targets for inhibiting OSCC progression [108]. Cancer drugs targeting SLC16A1–3 have been developed; for example, the SLC16A1 (MCT1) inhibitor AZD3965 was in a phase I clinical trial in patients with advanced solid tumors or lymphomas [120]. AZD3925 is currently in phase I/II clinical trials for hematopoietic tumors in Japan. AZD3965 has also shown promising results in slowing tumor growth in mouse models when combined with simvastatin, suggesting that SLC16A1 inhibitors can be effectively combined with other drugs to enhance anticancer effects [108,121].

Genetic analysis of SLC transporters has revealed significant variation among individuals and ethnic groups, and this genetic diversity may affect drug efficacy and toxicity. This could lead to personalized treatment approaches and could be an attractive target for precision medicine [59]. Epigenetic modifications such as DNA methylation also play a role in regulating the expression of SLC transporters in cancer, affecting drug response and tumor growth. This highlights the potential for targeting epigenetic changes to modulate SLC transporter activity in cancer therapy [122]. It has also been suggested that some SLCs affect the immune response. The interaction of SLCs with the immune response in OSCC therapy should also be focused on [92]. Although significant progress has been made in our structural understanding of SLC transporters, there are still many aspects that have not been explored. The complexity of the transport mechanisms and the diversity of the substrates transported continue to challenge researchers. The development of more advanced computational models and experimental techniques will improve our understanding of the dynamics of SLC transporters and their interactions with drugs and may further expand their potential as therapeutic targets.

## 5. Conclusions

The two major families of plasma membrane transporters, ABC transporters and SLC transporters, play pivotal roles in the uptake and excretion of various substances within the cell. It is known that drug excretion by ABC transporters affects therapeutic efficacy. Researchers have studied inhibitors of ABC transporters to counteract drug resistance, but most have limited clinical application due to major challenges of toxicity to normal cells and limited efficacy. On the other hand, SLC transporters have not been as well studied in cancer as ABC transporters. SLC transporters contribute significantly to the metabolic upregulation of cancer cells due to their ability to transport nutrients and affect therapeutic efficacy due to the uptake of drugs. In recent years, the importance of SLC transporters has become increasingly recognized, and their relevance to cancer has been increasingly reported. Some SLC transporter inhibitors are expected to find clinical application due to their inhibition of cancer growth and efficacy in combination with other drugs.

Advances in genomic diagnostics have revealed limited expression of drug resistance attributable to the ABC transporter, which is thought to be responsible for the variability and low efficacy of the inhibitors studied. Genetic analysis of SLC transporters has also revealed significant variation among individuals and ethnic groups, and this genetic diversity may affect drug efficacy and toxicity. Advances in genomic diagnostics can help identify patient-specific transporter expression, leading to more personalized and effective treatment strategies. In oral cancer, the more advanced the cancer type, the less research has been conducted on transporters, especially SLC transporters. Future research should focus on characterizing the expression patterns of SLC transporters in oral cancer and developing personalized medicine approaches to optimize drug delivery and therapeutic efficacy considering these patterns.

## Figures and Tables

**Table 2:** List of solute carrier (SLC) transporters expressed in oral cancer.

Family Name	Approved Symbol	Alias Symbol	HGNC ^#^ ID	References
SLC1 (High-affinity glutamate and neutral amino acid transporter family)				
	SLC1A5	AAAT; ASCT2	10943	[65,66,67,68,69]
SLC2 (Facilitative glucose transporter family (previous name GLUT))				
	SLC2A1	GLUT-1; DYT18; DYT19	11005	[57,70,71,72,73,74,75,76,77,78]
	SLC2A2	GLUT-2	11006	[70,79]
	SLC2A3		11007	[4,80,81,82,83]
	SLC2A4		11009	[4,70,79]
	SLC2A5 *		11010	[61,84]
	SLC2A13	HMIT	15956	[4,85]
SLC3 (Heavy subunits of the heteromeric amino acid transporters)				
	SLC3A2	4T2HC; 4F2	11026	[62,86,87]
SLC5 (Sodium-glucose cotransporter family)				
	SLC5A1	SGLT-1	11036	[4,57]
SLC6 (Na^+^- and Cl^−^-dependent neurotransmitter transporter family)				
	SLC6A3 *	DAT	11049	[88,89,90]
	SLC6A8 *	CRTR; CT1	11055	[91]
	SLC6A14 *	CRTR; CT1	11047	[92]
SLC7 (Cationic amino acid transporter/glycoprotein-associated family)				
	SLC7A5	LAT1	11063	[4,62,65,68,69,82,87,93]
	SLC7A11	xCT	11059	[68,94,95]
SLC16 (Monocarboxylate transporter family)				
	SLC16A3	MCT3; MCT4	10924	[4,59,63,82]
SLCO (Organic anion transporting family (previous family name SLC21))				
	SLCO1A2	OATP; OATP1A2	10956	[62]
	SLCO1B3	OATP8; OATP1B3	10961	[62]
	SLCO2A1	PGT; OATP2A1	10955	[96]
	SLCO2B1	OATP-8; OATP2B1	10962	[97]
SLC22 (Organic cation/anion/zwitterion transporter family)				
	SLC22A3	OCT3; EMT	10967	[96,98]
	SLC22A4	OCTN1	10968	[93,96]
	SLC22A5	OCTN2	10969	[93,96]
	SLC22A16	CT2; OAT6	20302	[96]
SLC25 (Mitochondrial carrier family)				
	SLC25A32	MFTC	29683	[87]
SLC29 (Facilitative nucleoside transporter family)				
	SLC29A1 *	ENT1	11003	[62]
SLC30 (Zinc efflux family)				
	SLC30A1 *	ZRC1 (ZNT1)	11012	[4,99,100]
SLC31 (Copper transporter family)				
	SLC31A1 *	CTR1	11016	[87]
SLC34 (Type II Na+ Phosphate cotransporter family)				
	SLC34A2 *	NAPI-3B; NaPi-2b	11020	[87]
SLC38 (System A & N, Na^+^-coupled neutral amino acid transporter family)				
	SLC38A1	SNAT1; ATA1	13447	[59,101]
SLC39 (Metal ion transporter family)				
	SLC39A1 *	ZIP1	12876	[99,100]
	SLC39A4 *	ZIP4	17129	[87]
	SLC39A13 *	ZIP13	20859	[87]
SLC53 (Phosphate carriers (phosphate exporter)				
	XPR1	SLC53A1; SYG1	12827	[82,87]

* Not reported in OSCC and HNSCC. ^#^ Human Gene Nomenclature Committee.

## Abbreviations

The following abbreviations are used in this manuscript:

ABC	ATP-binding cassette
SLC	solute carrier
OSCC	oral squamous cell carcinoma
HNSCC	head and neck squamous cell carcinoma
HPV	human papilloma virus
NBDs	nucleotide-binding domains
5-FU	5-fluorouracil
TMDs	transmembrane domains
MDR 1	multidrug resistance 1
BCRP	breast cancer resistance protein
CSCs	cancer stem cells
FACS	fluorescence-activated cell sorting
SP	side population
IL	interleukin
STAT3	signal transducer and activator of transcription 3
EMT	epithelial-mesenchymal transition
NF-κB	nuclear factor kappa B
OCT	organic cation transporter
MCT	monocarboxylic amino acid transporter
TCGA	The Cancer Genome Atlas
HPA	Human Protein Atlas
MTF	mitochondrial folate transporter
